# Familial aggregation of albuminuria and arterial hypertension in an Aboriginal Australian community and the contribution of variants in *ACE* and *TP53*

**DOI:** 10.1186/s12882-016-0396-2

**Published:** 2016-11-21

**Authors:** David L. Duffy, Stephen P. McDonald, Beverley Hayhurst, Sianna Panagiotopoulos, Trudy J. Smith, Xing L. Wang, David E. Wilcken, Natalia L. Duarte, John Mathews, Wendy E. Hoy

**Affiliations:** 1 Genetic Epidemiology Laboratory, QIMR Berghofer Institute of Medical Research, 300 Herston Rd, Brisbane, 4006 Australia; 2 The Queen Elizabeth Hospital, Adelaide, Australia; 3 Cradle Coast Authority, Tasmania, Formerly Menzies School of Health Research, Darwin, Australia; 4 Department of Medicine, University of Melbourne, Melbourne, Australia; 5 Menzies School of Health Research, Darwin, Australia; 6 Department of Genetics, Southwest Foundation for Biomedical Research, San Antonio, Texas Australia; 7 Cardiovascular Genetics Department, Prince of Wales Hospital, Sydney, Australia; 8 Melbourne School of Population and Global Health, University of Melbourne, Melbourne, Australia; 9 Centre for Chronic Disease, The University of Queensland School of Medicine, Brisbane, Australia; 10 Centre for Chronic Disease, Central Clinical School, Royal Brisbane Hospital, Queensland, 4029 Australia

**Keywords:** Albuminuria, Epidemiology, Genetics, Heritability

## Abstract

**Background:**

Aboriginal Australians are at high risk of cardiovascular, metabolic and renal diseases, resulting in a marked reduction in life expectancy when compared to the rest of the Australian population. This is partly due to recognized environmental and lifestyle risk factors, but a contribution of genetic susceptibility is also likely.

**Methods:**

Using results from a comprehensive survey of one community (*N =* 1350 examined individuals), we have tested for familial aggregation of plasma glucose, arterial blood pressure, albuminuria (measured as urinary albumin to creatinine ratio, UACR) and estimated glomerular filtration rate (eGFR), and quantified the contribution of variation at four candidate genes (ACE; *TP53*; *ENOS3*; *MTHFR*).

**Results:**

In the subsample of 357 individuals with complete genotype and phenotype data we showed that both UACR (h^2^ = 64%) and blood pressure (sBP h^2^ = 29%, dBP, h^2^ = 11%) were significantly heritable. The *ACE* insertion-deletion (*P =* 0.0009) and *TP53* codon72 polymorphisms (*P =* 0.003) together contributed approximately 15% of the total heritability of UACR, with an effect of ACE genotype on BP also clearly evident.

**Conclusions:**

While the effects of the *ACE* insertion-deletion on risk of renal disease (especially in the setting of diabetes) are well recognized, this is only the second study to implicate p53 genotype as a risk factor for albuminuria - the other being an earlier study we performed in a different Aboriginal community (McDonald et al., J Am Soc Nephrol 13: 677-83, 2002). We conclude that there are significant genetic contributions to the high prevalence of chronic diseases observed in this population.

**Electronic supplementary material:**

The online version of this article (doi:10.1186/s12882-016-0396-2) contains supplementary material, which is available to authorized users.

## Background

Chronic diseases such as diabetes mellitus, hypertension, ischaemic heart disease, and chronic renal failure are far more common among Aboriginal Australians than they are in Australians of European descent [2–5]. This is especially true of Aboriginal communities in the “Top End”, the tropical northern part of the Northern Territory. In one community, we have demonstrated that these findings can be explained, at least in part, by the presence of a number of environmental and behavioural risk factors for these diseases. Notably, high rates of childhood Group A streptococcal infection and post-streptococcal glomerulonephritis (up to 28% of one sample under the age of 30 years [6]) were associated with later development of micro- and macro albuminuria [7, 8]. Obesity, alcohol and tobacco overuse were also commonly present.

However, we were also intrigued by the fact that end stage renal disease and albuminuria seemed to aggregate within particular families in these communities [9], an observation that might reflect segregation of disease genes. A number of risk loci have been characterised in other ethnic groups: strikingly, the five-fold difference in incidence of renal diseases between African and European descended populations is largely due to two coding variants in the *APOL1* gene [10–13]. Using data from another Aboriginal community we have previously shown that variants in two other much studied genes, the *ACE* insertion-deletion (indel) and TP53*R72P polymorphisms, are associated with albuminuria and hypertension [1]. *ACE* encodes angiotensin I converting enzyme, and *TP53,* the key tumor suppressor protein p53. The latter is involved in regulating responses to DNA damage, whether repair or programmed cell death.

We have a particular interest in albuminuria, as measured by the urinary albumin to creatinine ratio (UACR), as it is easily measured in the field, and is a good predictor not just of subsequent development of end stage renal disease, but also of cardiovascular disease morbidity and mortality in both diabetics and nondiabetics [4, 5, 14–17].

In the present paper, we quantify the effects of these *ACE* and *TP53* gene variants in a much larger Aboriginal Australian sample. Family based analysis is used to compare these effects to those of unmeasured genetic risk factors acting in this population on UACR, arterial blood pressure, and plasma glucose.

## Methods

### Study population

As described elsewhere [2, 4–8], a number of surveys (interviews and clinical examinations) and an intervention program focusing on renal and cardiovascular diseases have been carried out in a remote Aboriginal Australian community living on two islands situated off the coast of the Northern Territory, north of Darwin.

All participants completed a questionnaire recording basic demographic information, family relationships, and medical history. Height, weight and hip and waist circumferences were recorded. BP was measured by a single observer using a mercury sphygmomanometer. Urine and blood were collected for routine biochemical screening. In a subset of individuals with elevated spot plasma glucose levels, a 2 h oral glucose tolerance test was performed. The protocol was approved by the institutional ethics committee and written informed consent was obtained from all participants.

### Laboratory methods

Urinary albumin and creatinine levels in a spot urine collection were used to calculate the albumin:creatinine ratio (UACR), a measurement known to strongly correlate with 24 h urinary albumin excretion. Repeat UACR measurements were available for only 63 out of 357 individuals, with a test-retest correlation for log transformed UACR of 0.97. We estimated eGFR from serum creatinine level using the MDRD Study equation. Whole blood (4 ml) for DNA analysis was collected into EDTA tubes, stored at 4 °C until DNA extraction. DNA specimens were available for 401 subjects. Genotyping was carried out at 6 polymorphisms in four candidate genes: *NOS3 (*endothelial nitric oxide synthase*;* rs2070744, rs1799983, i4 VNTR), *TP53* (rs1042522), *MTHFR* (methylenetetrahydrofolate reductase; rs1801133) and *ACE* (rs4646994) as previously described [1]. These variants were selected because of previously reported associations with diabetes and diabetic nephropathy, or cardiovascular disease (eg [18–22]), and all were polymorphic in the present population (Additional file 1: Table S1).

### Statistical methods

Standard statistical analyses were carried out using the R statistical package [23]. The mgcv [24] and SemiPar packages [25] were used to investigate nonlinear covariate effects in penalized spline mixed models (with family-specific intercepts). The family based variance components analyses were carried out using the Sib-pair program [26], MENDEL 5.7 [27] and WOMBAT [28].

Urinary albumin to creatinine ratio was log transformed for subsequent analyses (logACR). Since 162 individuals had a 2 h OGGT plasma glucose measurement, but 197 a fasting plasma glucose, we have analysed the latter, using a log transformation. Familial aggregation of quantitative traits such as logACR and systolic (sBP) and diastolic (dBP) blood pressure were carried out using a mixed effects model (variance components model). In this analysis we relate the degree of genetic relationship between individuals to their similarity in phenotypes. The random effects were assumed to be multivariate normally distributed, and this was tested by evaluation of model residuals. We utilized the usual biometrical decomposition [29] of the variance into components due to additive genetic random effects (correlated among family members) and residual environmental effects. The heritability is a commonly quoted summary statistic from this type of analysis, and is the proportion of the phenotypic variance of the trait in this population due to genetic effects. Since we have not modeled household effects, it is not possible to disentangle the effects of household and familial environment from genetic effects, and this should be borne in mind when examining the heritability estimates. Fixed effects of age, weight, height, sex and measured genotype were included in the genetic models.

## Results

Clinical data are available for a total of 1350 individuals from the community, more than 80% of community members of 5 years of age and over. A subsample of 401 individuals were genotyped, chosen on the basis of their ranges of urine ACR being representative of that of the whole community. Pedigrees were successfully constructed for 357 of these individuals. There were a total of 771 pedigree members (including unphenotyped “connecting” individuals), with a mean pedigree size of 5.7 individuals (range 3-43), and the deepest pedigree spanned 5 generations. Slightly more males than females were examined (184 M, 173 F), and ages ranged from 18 to 76 years (see Table 1).

**Table 1:** Descriptive statistics for key phenotypes in individuals with measured UACR

Variable	All (*N =* 357)	Males (*N =* 184)	Female (*N =* 173)
Median Age (range)	34.6 (18.0–76.4)	32.5 (18.0–73.2)	36.5 (18.0–76.4)
Geometric mean UACR (range)	5.3 (0.1–605.8)	6.8 (0.2–605.8)	4.3 (0.1–466.7)
Percentage UACR > 33 g/mol	25.5%	22.8%	28.3%
Median BMI (range)	22.7 (14.2–43.5)	22.1 (14.2–39.1)	23.1 (14.6–43.5)
Median serum creatinine	81.0 (69–589)	91 (63–589)	69 (63–238)
Percentage smokers	26.3%	21.3%	31.7%
Median fasting glucose (range) *N =* 197	4.7 (3.2–18.1)	4.6 (3.2–17)	4.8 (3.6–18.1)
Percentage fasting glucose > 5.5	22.8%	15.9%	31.1%
Median systolic BP (range) *N =* 347	120.0 (84–170)	124 (84–170)	114 (89–170)
Median diastolic BP (range)	72 (39–130)	76 (42–130)	70 (39–112)

The subsample for the genetic analyses did not differ significantly from the complete sample in terms of sex ratio, rates of diabetes, blood pressure or UACR. For example, 40% of the complete sample aged 40-49 years were macroalbuminuric (UACR > 33 g/mol) versus 39% of the familial subsample. This rate can be compared to the overall albuminuria rate of 5% in the 1.8 million individuals (mean age 48) from 26 general population samples described by Nitsch and coworkers [17].

**Fig. 1:**
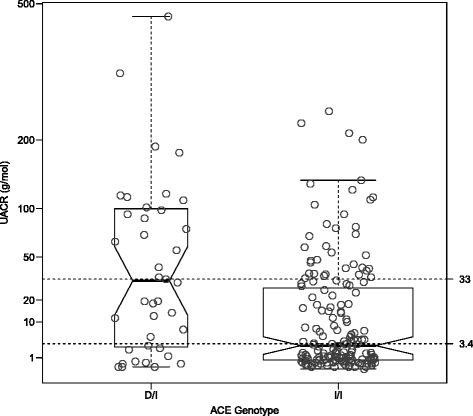
Urinary albumin:creatinine ratio versus ACE insertion-deletion genotype in a sample of Aboriginal Australians. Lines at UACR of 3.4 and 30 g/mol represent suggested diagnostic thresholds for micro- and macroalbuminuria. The notches represent 95 % confidence intervals around the median of each group

**Fig. 2:**
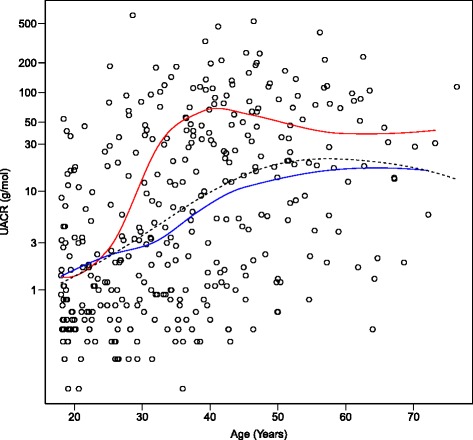
Effect of ACE insertion-deletion genotype on cross-sectional relationship between age and UACR. *Upper curve* is D/I genotype group; *lower*, the I/I genotype group; the dotted line is the overall curve (Flattening of D/I curve after the age of 40 may reflect selective mortality, but there may be other cohort effects acting)

**Fig. 3:**
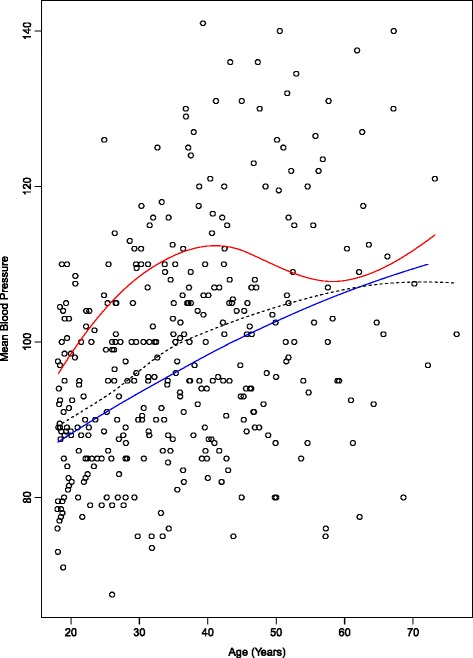
Effect of ACE insertion-deletion genotype on cross-sectional relationship between age and diastolic blood pressure. *Upper curve* is D/I genotype group; *lower*, the I/I genotype group; the* dotted line* is the overall curve

**Fig. 4:**
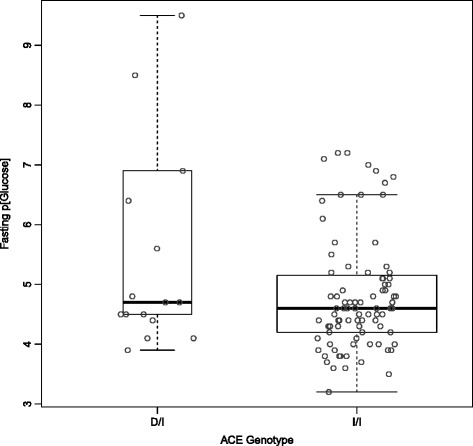
Effect of ACE insertion-deletion genotype on fasting plasma glucose

There were strong correlations between the various different traits (Table 2, Figs. 1, 2 and 3). Unsurprisingly, both logACR and BP were correlated with age. There were significant effects of genotype at the *ACE* indel and *TP53* codon72 polymorphisms on multiple clinically relevant phenotypes (Additional file 1: Table S2). Microalbuminuria (defined as 3.4-33 g/mol) was increased in ACE D/I carriers compared to I/I carriers (RR = 1.4, and see Fig. 1), and macroalbuminuria (>33 g/mol) even further so (RR = 2.2), with 47.5% of D/I carriers macroalbuminuric, but only 21% of the I/I genotype (*P =* 0.001). The effects of ACE genotype on UACR were most obvious after the age of 30 years (see Fig. 2). The D/I carriers also had higher systolic (130.4 versus 119.2 mm Hg) and diastolic blood pressures (83.3 versus 70.7 mm Hg) than I/I carriers (Fig. 3; Additional file 1: Figure S3 and S4), and were four times more likely to be diabetic (22% versus 5%, and see Fig. 4).

**Table 2:** Pearson correlations between key phenotypes and covariables (Pairwise *N =* 327-357)

	Sex	Age	Ht	Wt	BMI	Cr	eGFR	ACR	dBP	sBP
Sex	1.00									
Age	-0.12	1.00								
Height	**0.70**	-0.01	1.00							
Weight	0.21	0.15	**0.38**	1.00						
BMI	-0.09	0.17	-0.03	**0.91**	1.00					
Creatinine	**0.32**	0.14	0.26	**0.31**	0.21	1.00				
eGFR	-0.27	**-0.38**	-0.10	**0.45**	**0.53**	**-0.47**	1.00			
logACR	-0.11	**0.45**	-0.01	0.29	**0.30**	0.19	-0.04	1.00		
dBP	0.19	**0.32**	0.25	**0.43**	**0.35**	**0.32**	-0.07	**0.42**	1.00	
sBP	0.25	**0.36**	0.24	**0.33**	0.25	0.27	-0.14	**0.33**	**0.61**	1.00

Bolding denotes a correlation that is statistically significantly different from zero (*P <* 0.05)

Estimated glomerular filtration rate (eGFR, available for 335 individuals) was nonlinearly related to logACR, as well as to weight. It did not show any significant relationship to *ACE* or *TP53* genotype.

The multivariate genetic variance components analysis of logACR and BP using MENDEL found that the correlation between these traits was almost completely genetic in nature, with little evidence of a unique environmental (ie intraindividual nongenetic) correlation (Table 3). The heritability of logACR was 64%, while that of sBP 24% and dBP only 11%. The multivariate adjusted effects of *ACE* genotype (D/I versus I/I) were 6.8 mm Hg of systolic blood pressure, 8.2 mm Hg of diastolic blood pressure, and a 2.7-fold increase of UACR (see Table 4). The effect of the TP53*P72 allele on UACR was approximately half that of the ACE*D allele: 1.6-fold. We estimate that approximately 9% of the genetic variance of logACR is due to *ACE*, and 5% due to *TP53*. In contrast to this, variance components analyses of both eGFR and serum creatinine levels estimated the heritabilities of those traits as not significantly different from zero.

**Table 3:** Additive genetic and environmental correlations and heritabilities (main diagonal) from mixed model of BP, logACR, and log transformed fasting plasma glucose (logFPG)

	Additive Genetic				Non-familial Environmental			
	sBP	dBP	logACR	logFPG	sBP	dBP	logACR	logFPG
sBP	0.26				0.74			
dBP	0.74	0.11			0.47	0.88		
logACR	0.22	0.81	0.55		0.22	0.21	0.45	
logFPG	-0.73	-0.15	0.38	0.06	0.24	0.16	0.16	0.94

**Table 4:** Parameter estimates for fixed effects regression part of mixed model for logACR, BP and log fasting plasma glucose

Trait	Predictor	Estimate	ASE	Wald *P*-value
logACR	Male Sex	-0.114	0.0830	
	Age	0.029	0.0030	
	Weight	-0.0007	0.0009	
	ACE*D	0.47	0.13	0.0003**
	TP53*P	0.20	0.074	0.007**
sBP	Male Sex	10.5	1.6	
	Age	0.52	0.061	
	Weight	-0.0015	0.0017	
	ACE*D	6.8	2.5	0.006**
	TP53*P	-0.5	1.4	0.72
dBP	Male Sex	7.2	1.5	
	Age	0.38	0.056	
	Weight	-0.0017	0.0016	
	ACE*D	8.2	2.2	0.0005**
	TP53*P	-0.6	1.2	0.62
Log Fasting plasma glucose	Male Sex	0.031	0.022	
	Age	0.0029	0.00067	
	Weight	0.0020	0.00053	
	ACE*D	0.050	0.026	0.019*
	TP53*P	0.0019	0.013	1.0

* highlights a *P*-value in the range 0.01-0.05

** highlights a P-value <0.01

## Discussion

We have now reported a number of studies involving this Australian Aboriginal community [2, 4–10], documenting much higher rates of diabetes mellitus, arterial hypertension, albuminuria and renal disease than are seen in the general Australian population. In the current work, we confirm the clinical impression that both albuminuria and renal insufficiency seems to aggregate within particular families from this community. Because of the correlation between our measure of albuminuria (logACR) and blood pressure, we have carried out multivariate genetic analyses. These found that logACR was strongly heritable, while BP less so. The correlation between logACR and BP was found to be largely genetic in nature.

We further showed that approximately 14% of the genetic variances of logACR and of BP were explained by polymorphisms at two loci, *ACE* and *TP53*. A number of studies have implicated the *ACE* insertion/deletion polymorphism in risk of diabetic nephropathy. Mooyart and coworkers’ [30] meta-analysis found 42 studies of ACE, giving an overall odds ratio for diabetic nephropathy of 1.24 (95% CI 1.12–1.37), for the deletion (D allele), but comment that the effects are more marked in Asian than European populations. Their point estimates agree well with those from the large Diabetes Control and Complications Trial (DCCT) [31], where the I/I genotype hazard ratio was 0.7 for microalbuminuria and 0.6 for severe nephropathy. The effect sizes in the present study are roughly comparable to the latter (we observed 0.71 and 0.45). Most studies have not found a relationship between ACE genotype and essential hypertension. For example, in a meta-analysis of 46 studies, Staessen and coworkers [32] report that although the ACE D allele clearly increased risk of coronary heart disease and stroke, the effects on blood pressure were not significant. In the present study, the D allele was significantly associated with increased blood pressure, which may reflect the high rates of renal disease in this community.

It is interesting to examine the frequency of the ACE indel across comparable populations to that in the current study (Table 5). Firstly, the frequency of the deleterious D allele in our sample is similar to that reported from other Australian Aboriginal samples. Secondly, it is less common than in most other world populations, with a suggestion of an African-European-Asian-Australian cline.

**Table 5:** Allele frequencies for the ACE insertion-deletion (rs4646994) and TP53 Arg72Pro (rs1042522) polymorphisms in multiple populations

	ACE		TP53	
Population	I	D	R (G)	P (C)
Aborigines: This Study	0.90	0.10	0.43	0.57
Aborigines: Groote Eylandt	0.88	0.12	0.45	0.55
Aborigines: Central Australia	0.91	0.09	-	-
PNG	0.75	0.25	0.16	0.84
Tamils	0.69	0.31	0.54	0.46
UK	0.49	0.51	0.69	0.31
Yoruba	0.35	0.65	0.36	0.64

The codon72 *TP53* polymorphism has not been commonly studied as a risk factor for renal disease. *TP53* is known to be important in acute renal injury via its role in regulation of apoptosis and cell survival, and inhibition of *TP53* expression by siRNA reduces the acute kidney injury following administration of aristolochic acid or renal ischemia-reperfusion in animal models, although longer term p53 inhibition may promote renal fibrosis in some species [33, 34]. The Arg72 p53 protein is more pro-apoptotic and tumour suppressive than the Pro72 version [35–37], and in the Hupki humanised mouse model increases rates of obesity and diabetes when fed a high fat diet [38]. In keeping with the latter, Burgdorf and coworkers’ metaanalysis of diabetes did find the R72 allele to increase risk of Type 2 diabetes by 6% [39], though there are no data for diabetic nephropathy *per se*. A relationship with BP, has also been reported [40], but we do not confirm this. There are large differences in frequency between continental populations, with the ancestral P72 allele common in Africa, and the R72 allele common in European and Asian populations (Table 5). In our study, frequency of the R72 allele was intermediate between Africa and Europe.

In view of the forgoing, this polymorphism is a plausible candidate, and the association found in our earlier study with UACR [1] is replicated here in a second Aboriginal community. That the P72 allele is the risk allele seems counterintuitive, but this may reflect the long term versus short term effect. Because the effect of the R72 allele on human diabetes risk is modest, it is not surprising that we did not detect any association in the present study with plasma glucose levels. Exploration of renal pathology in the humanized R72 prediabetic mouse model [38] may be of relevance.

For both the *ACE* and *TP53* variants, we should worry that the risk allele is actually a marker for European admixture or ancestry. One example of such ethnic confounding was for diabetes risk in Pima indigenous Americans [41]. Unfortunately, we did not have enough genotyped parent-offspring pairs to carry out a transmission-disequilibrium test (TDT) to definitively exclude this possibility, but point estimates from the TDT (not shown) matched that estimated by the variance components analysis. The observed direction of association is also inconsistent with this type of confounding, in that given the high prevalence of disease in the indigenous community, then European admixture should be protective.

Because we have not used an intrinsically informative design (such as an adoption or twin study), we cannot unequivocally differentiate between effects of other genes and unmeasured shared environmental factors as cause of the observed familial aggregation. One likely familial factor would be infection with, for example, group A streptococci. We did examine other factors such as birth weight (available for a subset of 110 individuals), but these did not aggregate strongly enough within families to act as a confounder [42, 43]. This means we also cannot address whether particular environmental exposures are exacerbating the effects of the candidate gene effects that we have demonstrated, given that the genotypic effect sizes are larger than those seen in other populations.

We found logACR to be a useful choice in comparison to other measures of renal function in terms of the linearity of its relationship with covariates such as age, and found it to be more heritable than covariate-adjusted serum creatinine level or glomerular filtration rate. In the present analysis we have used only cross-sectional (baseline) data. In previous longitudinal studies in this community, we have shown that logACR is an excellent predictor of change in GFR over time [4]. Aside from this, a number of mortality studies have now shown that albuminuria is a risk predictor independent of glomerular filtration rate [44]. Microalbuminuria occurs both in the context of systemic vascular endothelial dysfunction and in podocyte specific disorders, such as nephrotic syndrome, and so may represent either a common marker or a common pathway for different pathophysiological processes [45].

We have previously presented data showing how UACR increases in this population with age, flattening off by age 60 years (see Fig. 1 from Reference [14]). Fig. 2 in the present paper shows that the same shape relationship occurs for each *ACE* indel genotype, but that the I/D group rise faster, especially after the age of 30 years, but flattens off by age 45. This may represent effects of differential mortality or cohort effects – we have reported elsewhere that age-specific mortality rates may be improving in this community with time [46].

Several studies of the genetics of logACR have been published that we can compare our results to. These studies have concentrated on families of containing diabetic probands, but a common finding is that the heritability of logACR is the same in diabetic and nondiabetic family members. Fogarty et al. [47] performed a segregation analysis of UACR in 1269 individuals (630 diabetics) from 96 families, and concluded that there was strong familial aggregation, with a heritability of 0.25 (SE = 0.05). Langefeld et al. [48] report results from diabetes-concordant sib pair families (662 individuals in 310 families). The heritability of logACR was 0.42 (SE = 0.12), while that of GFR was 0.75 (0.10). Fox et al. [49] studied logACR in the Framingham families (1055 individuals in 330 families). The heritability of logACR was 0.20. The best linkage peak was lod = 2.22 on chromosome 8 (D8S1179). Krolewski et al. [50] also performed a genome-wide linkage scan of logACR in 63 diabetic families. The heritability of logACR was significant in both diabetic (h^2^ = 0.23, *P =* 0.0007) and nondiabetic (h^2^ = 0.39, *P =* 0.0001) family members, and the difference in genetic variance between diabetic and nondiabetic relatives was not statistically significant (*P =* 0.16). There was evidence of genetic linkage of logACR to regions on chromosome 22q (lod = 3.7) and chromosome 7q (lod = 3.1). In the 59 Caucasian families, an additional locus on 5q (lod = 3.4) also reached significance. Overall, the point estimates of the heritability for logACR from these studies are lower than in the present analysis, but within the 95% confidence interval of our estimate.

More recently, several genome-wide association studies have also been reported for UACR [51–53], as well as eGFR and ESRD [54–57]. Böger and colleagues [52] report a coding variant (rs1801239, I2984V) in the cubilin gene (*CUBN*) to be significantly associated with UACR. The second best SNP was rs13177732. For idiopathic membranous nephropathy, SNPs in *PLA2R1* (rs4664308) and *HLA-DQA1* (rs2187668) have been shown to be important in Caucasians, while multiple loci are risk factors for IgA nephropathy in Asians. It should be noted in this context that the dominant pathological findings in the Aboriginal population tend to glomerulomegaly followed by sclerosis [58]. The effects of the high penetrance African pathogenic alleles of *APOL1* (rs60910145, rs73885319, rs71785313) are restricted to non-diabetic nephropathies, that is focal segmental glomerulosclerosis, hypertensive end-stage renal disease, IgA nephropathy, HIV-related and lupus nephritis, but the high frequency of these alleles in Africa (37%) is strongly suspected to be due to Darwinian selection on trypanosomiasis susceptibility [59], a mechanism that will not act in this Australian population. Susceptibility to streptococcal infections may also be a genetic risk factor: mechanistically, Haapasalo et al [60] suggest interactions between bacteria and Complement Factor H (*CFH*) affect pathogeneticity, and found that a variant in *CFH* (rs1061170, CFH*Y402H) was weakly associated with severe streptococcal disease in European patients (speculatively, we would also highlight that mutations in *CFH5* are associated with C3 glomerulonephritis in Cypriots [61], though the mechanism in that condition is unknown). No information on frequency of any of these variants in Australian Aboriginal populations is available at present.

## Conclusions

In conclusion, we have adduced evidence of a significant genetic contribution to variation in risk of albuminuria in this Australian Aboriginal community, of a similar size to that seen in other populations. We demonstrated that two risk loci, the *ACE* insertion-deletion polymorphisms and the *TP53* codon72 each explain comparable proportions of this variance, but that the *ACE* D allele, at least, does not explain the high rates of renal dysfunction in this community, given that risk allele frequencies are lower than in other populations. The effect size of the *ACE* D allele was, however, slightly greater than that seen elsewhere.

## Additional file

Additional file 1: Supplementary figures and tables. (PDF 88 kb)

## Abbreviations

ACE: Angiotensin Converting enzyme gene
APOL1: Apolipoprotein L1
BMI: Body mass index
CFH: Complement factor H
CUBN: Cubulin gene
dBP: diastolic blood pressure
DCCT: Diabetes control and complications trial
eGFR: Estimated glomerular filtration rate
ESRD: End stage renal disease
lod: Decimal log odds ratio
logACR: Log-transformed urinary albumin to creatinine ratio
MTHFR: methylenetetrahydrofolate reductase gene
NOS3: Endothelial nitric oxide synthase gene
OGGT: Oral glucose tolerance test
P72: Proline at amino acid position 72 of a peptide
sBP: Systolic blood pressure
TDT: Transmission disequilibrium test
TP53: Tumour protein p53 gene
UACR: Urinary albumin to creatinine Ratio
VNTR: Variable number tandem repeat polymorphism
