# Supplementary material for: Familial aggregation of albuminuria and arterial hypertension in an Aboriginal Australian community and the contribution of variants in ACE and TP53
Source: BMC Nephrol. 2016 Nov 21;17:183. doi: 10.1186/s12882-016-0396-2 (PMC5117595; doi:10.1186/s12882-016-0396-2)

Supplementary Material

Supplementary Tables

Supplementary Table 1. SNP allele frequencies in present sample (maximum likelihood estimates and ASE).

Gene	SNP	Minor Allele (Other)	MAF (ASE)
ACE	rs4646994	D (I)	0.097 (0.015)
TP53	rs1042522	Arg (Pro)	0.427 (0.024)
NOS3	rs1799983 (G/T)	T (G)	0.011 (0.005)
	rs2070744	C (T)	0.113 (0.015)
	intron4 VNTR	B (A)	0.111 (0.015)
MTHFR	rs1801133	2 (1)	0.449 (0.024)

Supplementary Table 2. Likelihood ratio test based P-values simultaneously testing SNP association with systolic and diastolic blood pressure (sBP, dBP), log urinary albumin:creatinine ratio (UACR) and fasting plasma glucose (FPG), in a multivariate mixed model adjusting for age, sex, height and weight.

Gene	SNP	P-value
ACE	rs4646994	0.000770
TP53	rs1042522	0.017547
NOS3	rs1799983 (G/T)	0.538375
	rs2070744	0.938210
	intron4 VNTR	0.942033
MTHFR	rs1801133	0.360096

Supplementary Figures

Supplementary Figure 1. Semiparametric smooths for relationships of logACR and diastolic BP with age, sex, weight and genotype.

Supplementary Figure 2. Semiparametric smooths for relationship of eGFR with age, sex, height, BMI and genotype.

Supplementary Figure 3. Effect of ACE insertion-deletion genotype on cross-sectional relationship between age and mean blood pressure. Red curve is D/I genotype group; blue, the I/I genotype group; the dotted black line is the overall curve.

Supplementary Figure 4. Effect of ACE insertion-deletion genotype on cross-sectional relationship between age and diastolic blood pressure. Red upper curve is D/I genotype group; blue lower curve the I/I genotype group; the dotted black line is the overall curve.

Supplementary Figure 5. Effect of ACE insertion-deletion genotype on cross-sectional relationship between age and systolic blood pressure. Red upper curve is D/I genotype group; blue lower curve the I/I genotype group; the dotted black line is the overall curve.

Supplementary Figure 6. Effect of ACE insertion-deletion genotype on cross-sectional relationship between age and eGFR. Red curve is D/I genotype group; blue, the I/I genotype group; the dotted black line is the overall curve. Presumably, the presence of reserve capacity makes relationship with genotype less clearcut than with UACR, confirming the latter is a more sensitive measure.

Supplementary Figure 1. Semiparametric smooths for relationships of logACR and diastolic BP with age, sex, weight and genotype.

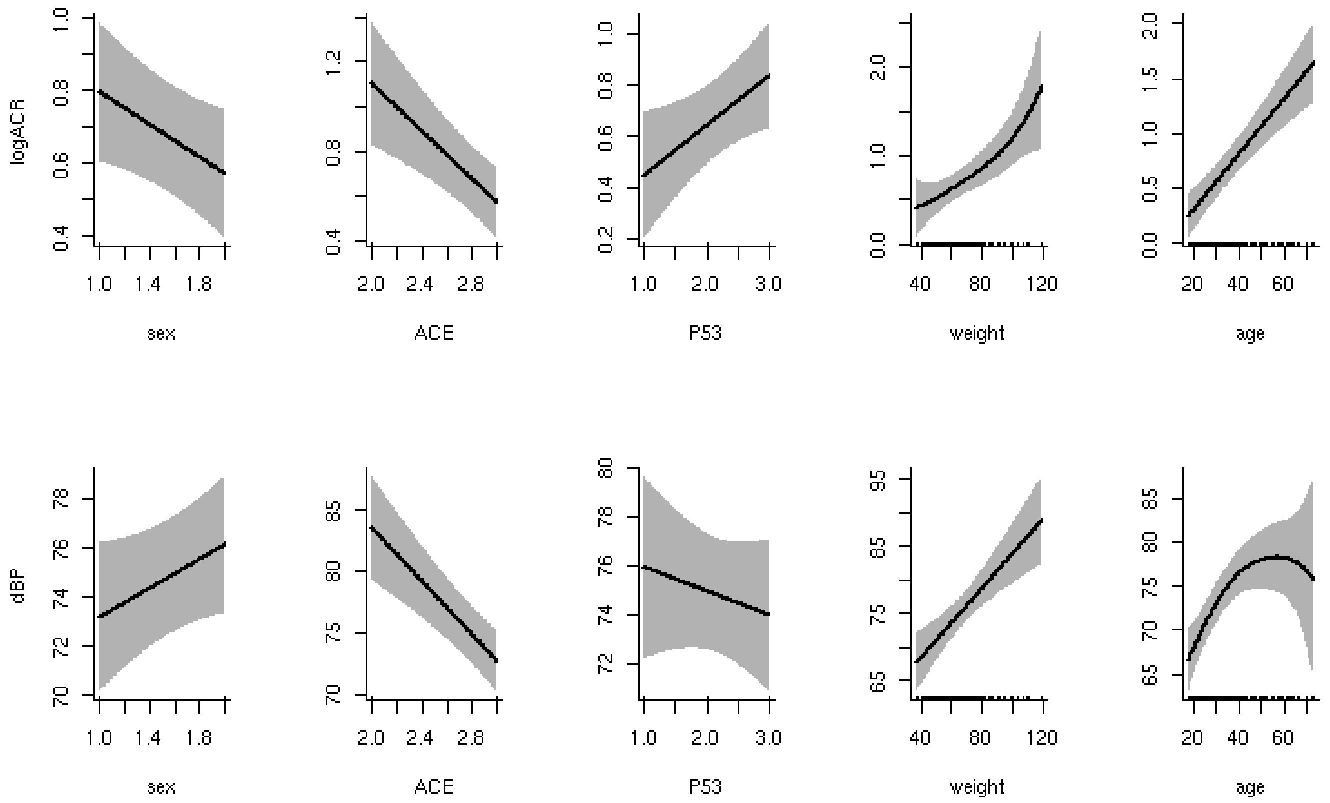

Supplementary Figure 2. Semiparametric smooths for relationship of eGFR with age, sex, height, BMI and genotype.

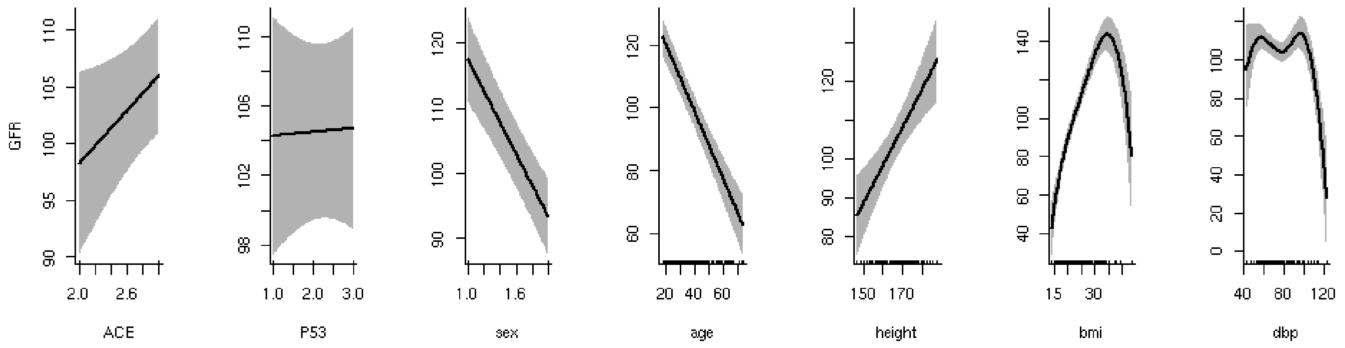

Supplementary Figure 4. Effect of ACE insertion-deletion genotype on cross-sectional relationship between age and diastolic blood pressure. Red upper curve is D/I genotype group; blue lower curve the I/I genotype group; the dotted black line is the overall curve.

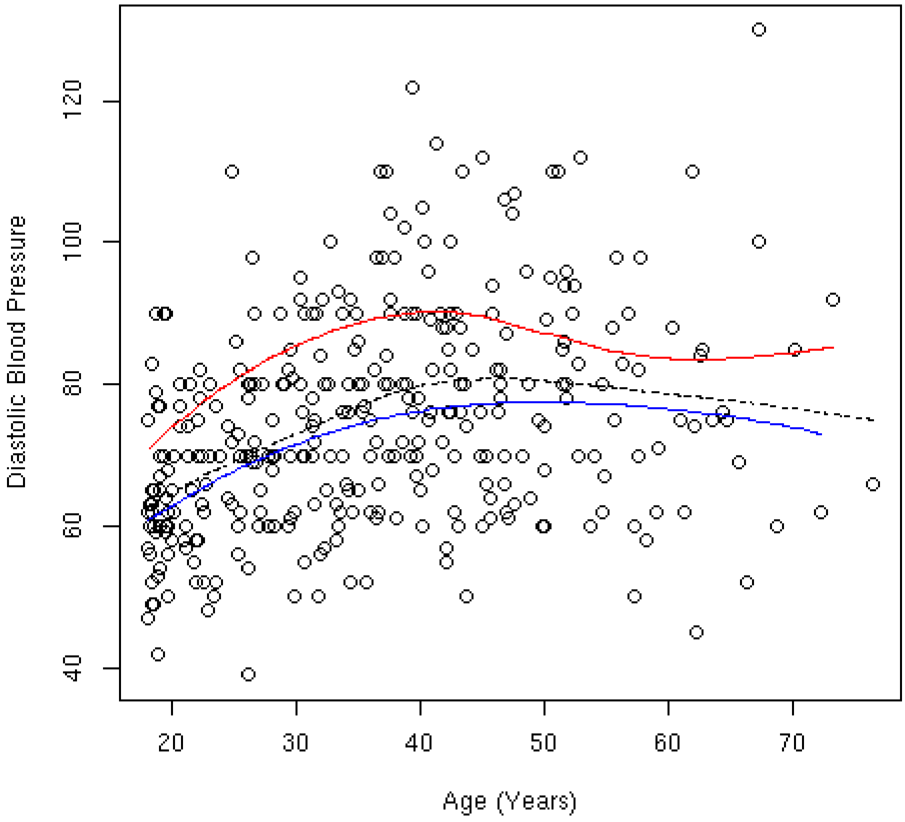

Supplementary Figure 5. Effect of ACE insertion-deletion genotype on cross-sectional relationship between age and systolic blood pressure. Red upper curve is D/I genotype group; blue lower curve the I/I genotype group; the dotted black line is the overall curve.

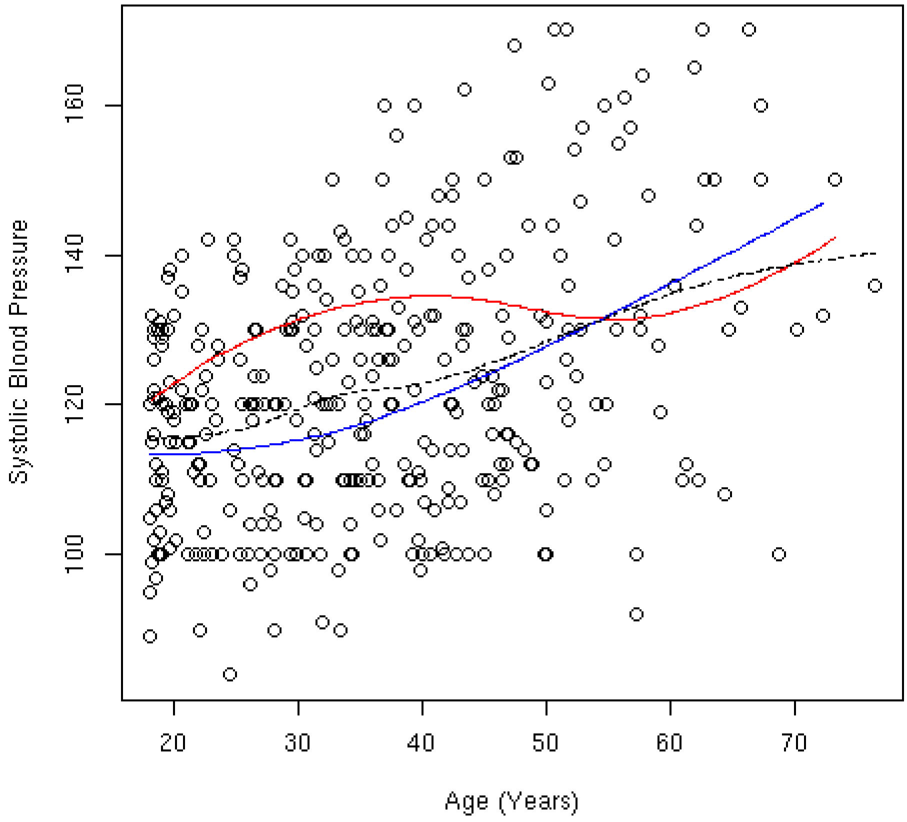

Supplementary Figure 6. Effect of ACE insertion-deletion genotype on cross-sectional relationship between age and eGFR. Red curve is D/I genotype group; blue, the I/I genotype group; the dotted black line is the overall curve. Presumably, the presence of reserve capacity makes relationship with genotype less clearcut than with UACR, confirming the latter is a more sensitive measure.

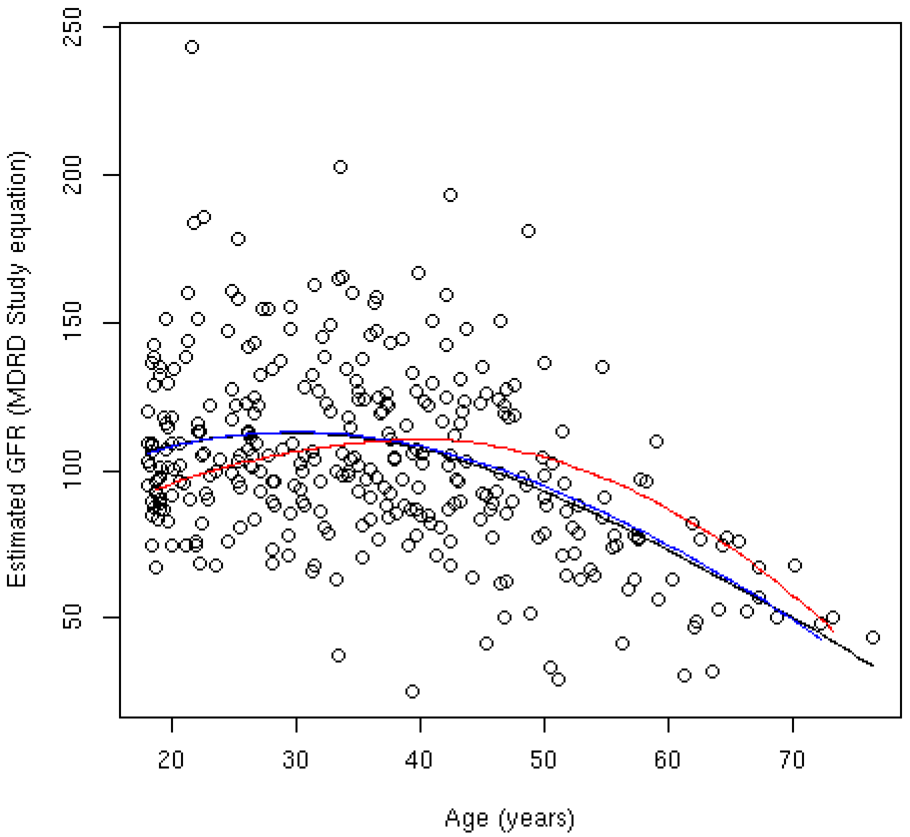

Supplement: Additional file 1: — Supplementary figures and tables. (PDF 88 kb) [file 12882_2016_396_MOESM1_ESM.pdf]
